# An AI based classifier model for lateral pillar classification of Legg–Calve–Perthes

**DOI:** 10.1038/s41598-023-34176-x

**Published:** 2023-04-27

**Authors:** Zafer Soydan, Yavuz Saglam, Sefa Key, Yusuf Alper Kati, Murat Taskiran, Seyfullah Kiymet, Tuba Salturk, Ahmet Serhat Aydin, Fuat Bilgili, Cengiz Sen

**Affiliations:** 1 Orthopedics and Traumatology, Bhtclinic İstanbul Tema Hastanesi, Nisantası University, Atakent Mh 4. Cadde No 36 PC, 34307 Kucukcekmece, Istanbul, Turkey; 2 Orthopedics and Traumatology, Istanbul University Istanbul Faculty of Medicine, Istanbul, Turkey; 3 Orthopedics and Traumatology, Bingol State Hospital, Bingol Merkez, Turkey; 4 Orthopedics and Traumatology, Antalya Egitim ve Arastirma Hastanesi, Antalya, Turkey; 5 Department of Electronics and Communication Engineering, Yildiz Technical University, Istanbul, Turkey; 6 Department of Informatics, Yildiz Technical University, Istanbul, Turkey

**Keywords:** Machine learning, Medical research

## Abstract

We intended to compare the doctors with a convolutional neural network (CNN) that we had trained using our own unique method for the Lateral Pillar Classification (LPC) of Legg–Calve–Perthes Disease (LCPD). Thousands of training data sets are frequently required for artificial intelligence (AI) applications in medicine. Since we did not have enough real patient radiographs to train a CNN, we devised a novel method to obtain them. We trained the CNN model with the data we created by modifying the normal hip radiographs. No real patient radiographs were ever used during the training phase. We tested the CNN model on 81 hips with LCPD. Firstly, we detected the interobserver reliability of the whole system and then the reliability of CNN alone. Second, the consensus list was used to compare the results of 11 doctors and the CNN model. Percentage agreement and interobserver analysis revealed that CNN had good reliability (ICC = 0.868). CNN has achieved a 76.54% classification performance and outperformed 9 out of 11 doctors. The CNN, which we trained with the aforementioned method, can now provide better results than doctors. In the future, as training data evolves and improves, we anticipate that AI will perform significantly better than physicians.

## Introduction

LCPD is an idiopathic avascular necrosis of the femoral head in the pediatric population. Treatment choice mainly depends on the prediction of the prognosis of femoral head sphericity and the congruency of the hip joint. The indication of containment treatment is determined by prognostic prediction. Several prognostic classification systems have been proposed for this purpose. Herring et al.^1^ described LPC in 1992 to predict the prognostic severity of disease at the fragmentation phase. According to LPC, the height of the lateral pillar is normal in group A. At least 50% of lateral pillar height was preserved in group B, and less than 50% of lateral pillar height was maintained in group C.

There are many studies in the literature about the interobserver reliability and prognostic value of LPC. Many studies reported moderate to high interobserver reliability and high prognostic value^2–11^, but as an opposing view, Agus et al.^12^ reported superior reliability for the Salter-Harris and Catterall classifications over the LPC. They found poor reliability in both intra- and inter-observer agreement in their study. Liggieri et al.^13^ stated that the degree of interobserver concordance was far from ideal for the three classification systems, including LPC, and advised complementary systems for staging. Some authors criticized this system by stating that there was a change between the first and last classifications^10,11,14^. Even according to Price^15^, LPC may only be predictive in retrospect after the opportunity for containment has passed, and there is still a need for the prospective indicators to guide treatment choice. Given the criticisms in the literature, as LPC may not be sufficient for staging in every patient, the prognosis may not be as expected. We anticipate that CNN will be a sharper prospective indicator in terms of classification and prognosis.

There are studies in the literature in which AI-based systems are used to solve the problems of proximal femur bone shape deformation in LCPD patients. The proximal femur bone shape deformity rates were determined using the patient's healthy femoral head in the study conducted by Memis et al. in 2021 using an image registration approach. Experimental studies using Magnetic Resonance Imaging (MRI) data from 13 patients suffering from LCPD have shown that the algorithm yields very promising results^16^. However, this technique is not useful for patients suffering from LCPD on both sides. In a subsequent study published in 2021 by the same research group, a method for rapidly and accurately extracting bilateral proximal femurs from bilateral hip joint MRI images utilizing random subsampling points was provided^17^. In the study conducted by Bugeja et al., a new automated three-dimensional (3D) pipeline for segmentation and measurement of cam volume, surface area, and height using MRI data from 97 patients suffering from femoroacetabular impingement (FAI) was provided. The proposed method in this study consists of two main parts: 3D U-net with focused shape modeling (FSM) for segmenting the proximal femur and identifying the cam bone mass and obtaining the anatomical information of the patient^18^. In another study using U-Net architecture, Memis et al. performed multiform proximal femur and femoral head bone segmentation from low-quality MRI sections with U-Net architecture^19^. These studies do not include any classification methods; they simply provide segmentation. In 2021, Memiş et al. used Support Vector Machine (SVM), one of the most widely used Machine Learning (ML) algorithms in the literature, to classify the Waldenstrom Stages of LCPD in MRI images and reported a 62% success rate^20^. These studies were all carried out using MRI scans. There is no research on LPC that uses AI with X-ray images. Also, in an other study on LCPD, the objective was not to stage the disease but rather to identify whether it was present or not, and it was claimed that the study was a great success^21^.

**Figure 1:**
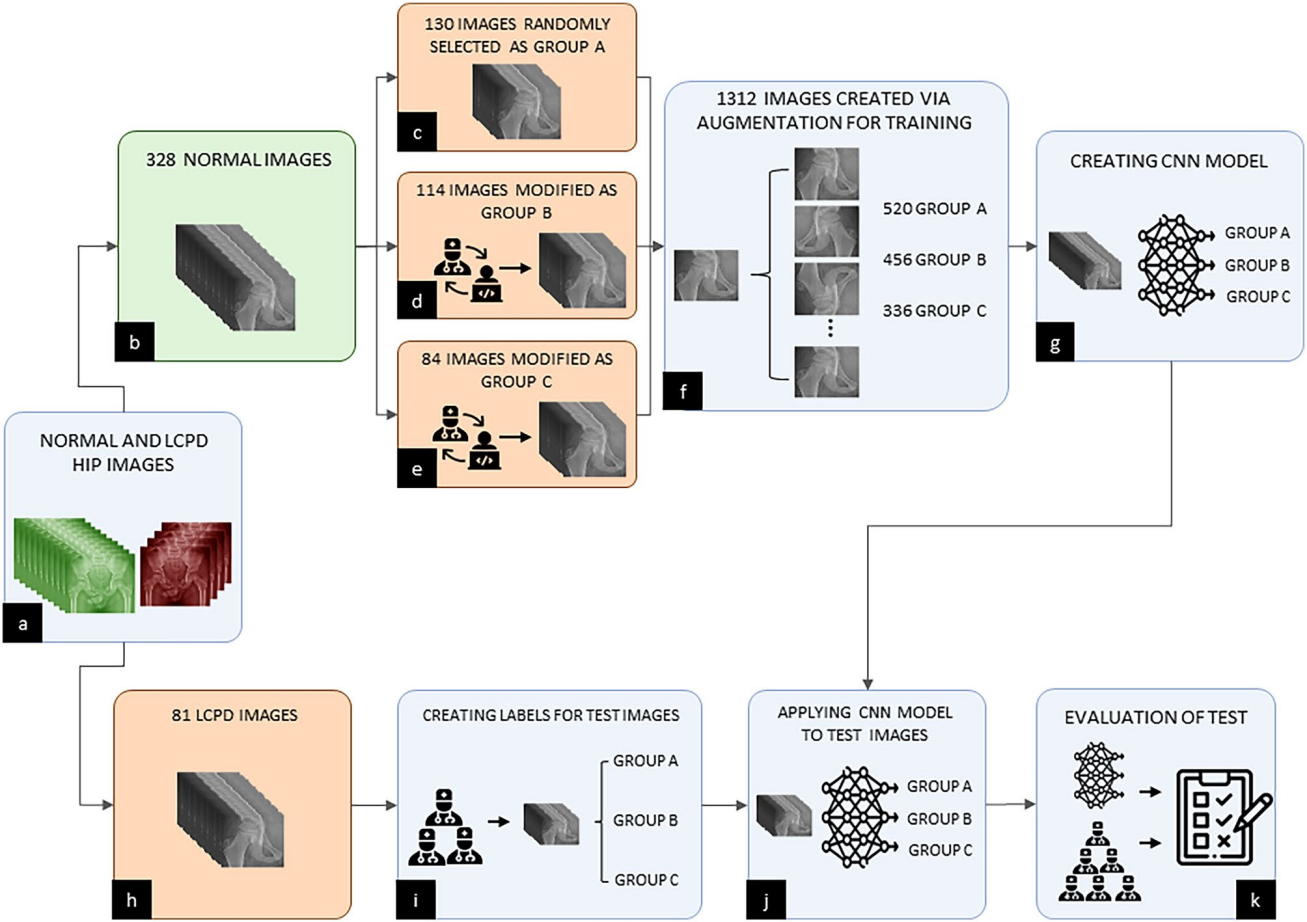
A flowchart of training and test progress (**a**) All normal and LCPD hips collected (**b**) Obtaining normal pediatric hip radiographs (328 images) that are representative of the real patient group in terms of age and gender distribution (**c**) Randomly selected 130 images from 328 normal images to create group A. (**d**, **e**) 114 images for group B and 84 images for group C modified by computer engineers under the guidance of six orthopedists (**f**) Augmenting the modified images to 1312 for training (520 in Class A, 456 in Class B, and 336 in Class C). (**g**) Designing CNN’s architecture and saving the CNN model for the test process (**h**) Obtaining real patients’ (LCPD) hip X-ray images (81 images). (**i**) Labelling LCPD patients’ hip radiographs with a unanimous decision by 6 senior orthopedists (Class A:29, Class B:31, Class C:21). (**j**) Applying the trained CNN model to test images and transferring the obtained classification results to the evaluation stage. (**k**) Testing the reliability of the proposed method and decision of the 6 doctors.

When the studies in the literature are examined, it is seen that the studies generally focus on the proximal femur bone shape segmentation. The reason for the small number of classification studies is the low number of patients suffering from LCPD and the failure to get the data required for training DL networks by researchers. Within the scope of this study, data that could represent LCPD patients were derived from modified data of healthy individuals, allowing researchers to train a DL network that could assist orthopedists with LPC. We aimed to show how CNN will give a result in terms of both reliability and percentage agreement in this regard. The flowchart, which provides information about the training and testing processes, is given in (Fig. 1).

Then, three more orthopaedists and two radiologists were added to the study, and the results of a total of nine orthopaedists and two radiologists were compared to those of CNN in terms of their classification accuracy.

The following sections provide more information about the proposed methods and the study's results.

## Materials and methods

### LCPD dataset

The study was approved by the local ethics committee of the Istanbul University Faculty of Medicine (No. 15/01/2020-003) and conducted in compliance with the 1964 Helsinki Declaration. The Ethics Committee of the Istanbul University Faculty of Medicine waived the requirement for informed consent because all data were anonymized and de-identified. Clinical records of LCPD patients on follow-up at two institutions between 2008 and 2016 were reviewed. One hundred eighteen patient hip radiographs were retrieved. Radiographs were excluded if they were not in the fragmentation phase. Hip radiographs taken in the neutral position from a distance of 120 cm in the full anteroposterior plane were used. Eighty-one radiographs were available for this study (72 males and 9 females, ages 4–12 years; mean age was 9.46; 45 right hips and 36 left hips). Only four males have bilateral LCPD. The time after onset was 4–6 weeks. At first, six orthopaedists with more than five years of pediatric orthopaedic experience made the classification individually. Then, a definitive list (a consensus list) was made by the unanimous decision of these six orthopaedists. We used the classic LPC and not the modified one because we did not have enough group b/c patient images to test. We had only four real patient images, which were classified as group b/c by doctors. This amount of data is not sufficient for researchers to obtain a reliable result. So, we decided to use classic LPC. Reliability tests were conducted using these six orthopaedists’ individual results and CNN’s results. After reliability tests, we added five more independent doctors, including two radiologists, for a percentage agreement test. They labeled 81 images individually as A, B, or C class. Finally, we compared the classification success rates of all of them using the percentage agreement test. Since all of the doctors were already familiar with LPC, none of them were given a tutorial at the start of this study.

**Figure 2:**
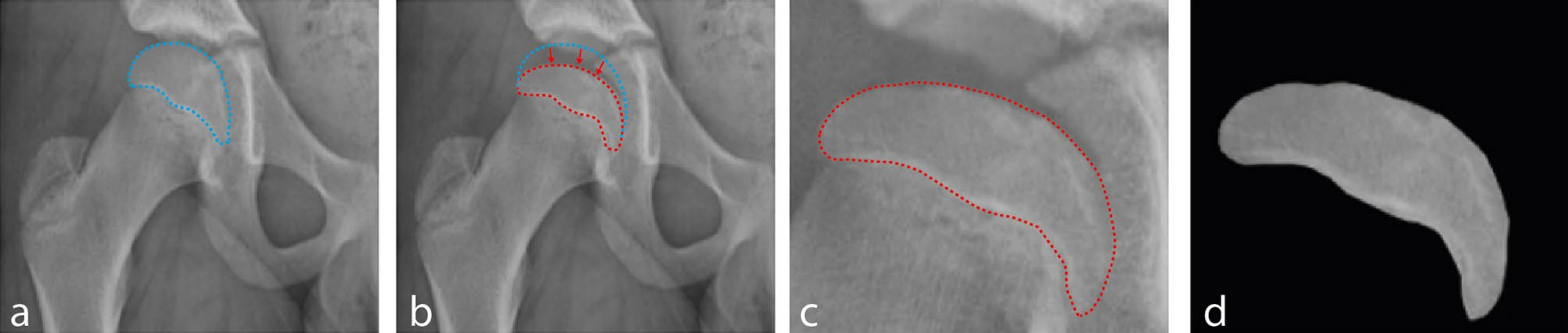
An illustration of how to produce a class B femoral head (**a**) Normal hip radiograph with a blue line encircling the femoral head (**b**) The height of the lateral column of the normal femoral head was reduced up to 50%. The head has been converted to group B. (**c**) The newly created head is highlighted in red. (**d**) The highlighted head is segmented from the image and assigned as the final training data.

The data set, described in Table 1, contains few samples due to the low prevalence of LCPD in the general population. A large number of images from various classes must be employed in order to train CNN architectures. The quantity of radiographs was insufficient for training the CNN architecture, so the authors devised a method for creating a training dataset. In this method, 328 radiographic images of healthy hips were obtained from patients between the ages of 4 and 12. 130 randomly chosen radiographs from the 328 images are labeled “group A”. Two computer engineers applied Adobe Photoshop® software to modify the femoral heads of the remaining 198 images under the guidance of six orthopedists. They produce 114 group B and 84 group C labeled images according to LPC (Fig. 2). Since modified images were created for training, all of the real LCPD radiographs were reserved to test the generated CNN model.

**Table 1:** Dataset description.

	Consensus list			
	Demographics	Grade A	Grade B	Grade C
Test data description				
Mean age (y.o)	9.46 (4–12 y.o.)			
Male	72	32	19	21
Female	9	4	3	2
Total	81	36	22	23
Training data description				
Mean age (y.o)	9.28 (4–12 y.o.)			
Male	290	116	102	72
Female	38	14	12	12
Total	328	130	114	84
After augmentation	1312	520	456	336

### Data Augmentation

In our study, there were 328 images of the femoral head for training, but the number of training samples was not enough to obtain acceptable results. According to Wong et al.^22^, augmentation has a significant impact on improving performance and reducing overfitting issues. Training data was augmented by horizontal flipping, zooming, and shearing of every image. At the end of the process, the number of training images increased to 1312 (520 for group A, 456 for group B, and 336 for group C).

### Preprocessing

**Figure 3:**
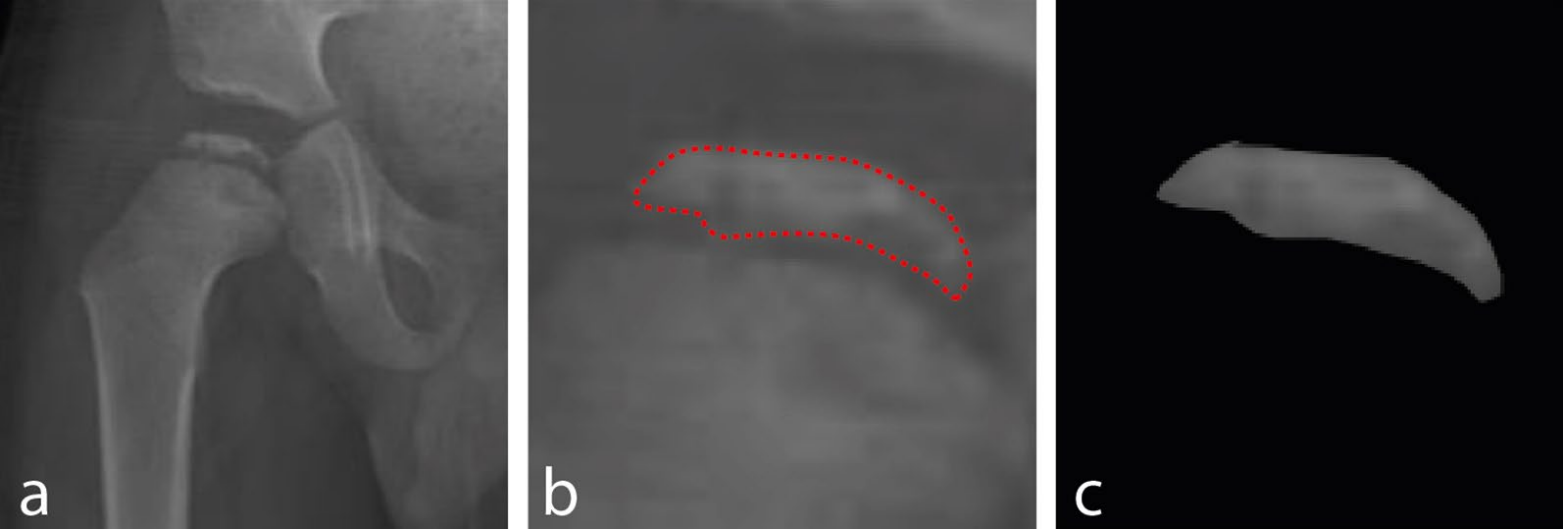
Obtaining the femoral head from the image (**a**) Hip radiograph of LCPD Group C (**b**) Femoral head pointed in red (**c**) The highlighted head is segmented from the image and assigned as the final test data.

The femoral heads that will be used in testing and training processes were segmented from real patient and modified hip radiographs in this section. During this process, the determination of femoral head boundaries and segmentation of these parts were performed under the supervision of six orthopedists (Fig. 3). After segmentation, other preprocessing approaches such as histogram equalization, sharpening, or morphological operations were applied to enhance the visual quality of the images. However, it was revealed that these preprocessing techniques had no significant impact on CNN's performance. So, it was decided not to use these preprocessing steps after segmentation because the CNN can work well enough without them.

### Deep learning (DL) and proposed CNN architecture

DL is a ML method that has recently attracted the attention of the medical sciences as well as many other fields. Unlike traditional ML methods, the DL algorithm learns features directly from the data via its multilayered structures^23^. To efficiently extract input data features, DL computes their internal parameters in the forward pass, then iteratively adjusts them during backpropagation. DL's forward and backpropagation procedures improve DL's learning capacity and provide a means to learn complex patterns in computer vision and other domains^24^. DL can be investigated through four main network structures. These are Deep Reinforcement Learning, Deep Boltzmann Machines, Recurrent Neural Networks (RNN), and CNNs. Systems that employ deep reinforcement learning interact with their surroundings and pick up knowledge from them^25^. Deep Boltzmann Machines are used for processing data by learning a probability density function over the inputs^26^. RNN are special in that they can operate on a series of vectors over time. This indicates that they can relate earlier knowledge to the current progress. With its several advantages, such as being more similar to the human visual processing system, CNN is employed in this study. It is widely used for the classification of images and is also proficient in learning and extracting features from 2D data^27^.

CNNs are a common type of deep learning neural network that is used for image classification applications. The network is composed of layers, each of which has a specific function. The fundamental layers of a CNN consist of the following:

*Convolutional layer* Convolution operations are carried out by this layer on the input image to aid in the feature extraction process. Each of the filters used by the convolutional layer on top of the image is designed to detect a certain feature in the image.

*Activation layer* This layer applies an activation function to the output of the convolutional layer, which is often the rectified linear unit (ReLU) function in image classification. The ReLU function enhances the network's capacity to learn complicated features by introducing non-linearity into the system.

*Pooling layer* Pooling is used to minimize the spatial dimensions of the feature maps, which reduces the computational cost of the network and increases the robustness of the features to slight changes in an object's position within an image.

*Fully connected layer* All the neurons in one layer are linked to the neurons in the next layer by this layer. Its purpose is to learn non-linear combinations of features from the previous layer.

*Output layer* This layer generates the network's final output, which could be a class label or a collection of class probabilities for an image classification task.

#### Training process

**Figure 4:**
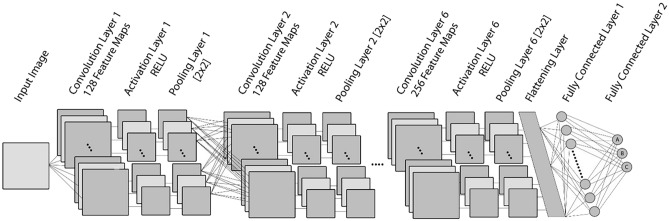
Proposed CNN Architecture for LPC of LCPD.

Figure 4 demonstrates the proposed neural network design, which consists of six convolutional layers, each followed by an activation and pooling layer. After the activation layers have normalized the output of the convolutional layers, the pooling layers use a maximum pooling operation with a kernel size of 2 × 2 to minimize the dimension of the data. Employing 3 × 3 filters, the first three convolutional layers generate 128 feature maps, while the following three convolutional layers generate 256 feature maps. The architecture also has a flattened layer and two fully connected layers. The activation function for all activation layers and the first fully connected layer is the ReLU function, which also serves as a solution to the vanishing gradient problem^28^. Only the last fully connected layer uses the Softmax activation function because the class of input is determined in this layer. Also, between the first and second fully connected layers, 0.2 ratio of randomly chosen neuron connections are dropped out to avoid overfitting^29^. While creating the CNN model, optimizers are implemented to minimize the error between the predicted output and the true output. Due to its capacity to manage the complexity of deep learning and its ability to adaptively adjust the learning rate for each parameter, the proposed network implements the Adaptive Moment Estimation (Adam) Optimizer with a learning rate of 0.001^30^. The selection of hyper-parameters such as number of convolution layers, activation functions, optimizer, dropout rate, learning rate and batch size used in this study were determined by grid search algorithm and the hyper-parameters that gave the best results were used in the study. Determining hyper parameters with the grid search algorithm is a widely used method in deep learning studies. Determining the hyper-parameters with an algorithm among various values enables the deep neural network to give results with higher performance in different problems and contributes to the creation of the model suitable for the problem. (Fig. 4).

Once the architecture of the network was established, images with dimensions of 224 × 224 × 3 were given to the first convolutional layer with a 32-batch size, and the training process was started with 1312 images labeled by six doctors. In the study, the epoch number was chosen as 1000, which is a large value to avoid the underfitting problem, and the early stopping criterion was applied to stop training to avoid overfitting when the validation value declined. The training was terminated automatically between 416 and 482 epochs, each epoch time was around 30 s, and the weights with the highest validation success were recorded. The early stopping criterion is optimized by using different patience values and patience value was chosen as 50 to prevent overfitting. Radiographic images of real patients, referred to as “test images”, are never used in the training process. They're only used throughout the testing process.

### Evaluation of metrics

After the training process of the model was completed, the network parameters obtained were recorded, and the network was made ready for experimental tests. Various evaluation metrics, such as accuracy, precision, specificity, recall (sensitivity), and F1 score, are used to evaluate the performance of the trained CNN during the tests. Accuracy is the most common evaluation metric for CNN classification systems because it is easy to understand and interpret. However, when dealing with unbalanced data, the F1 score is an effective option for evaluating the classification model. We also demonstrate the other evaluation metrics to support our classification model results. The accuracy rate of the system was 83.33% on validation and 76.54% on test images; the F1 score of the system on test images is 77.13% according to the consensus list.

$$Accuracy=\frac{\sum_{i}^{Total Class Number}{TP}_{i}+{TN}_{i}}{Total Observations}$$

$$Precision=\frac{\sum_{i}^{Total Class Number}{TP}_{i}}{({TP}_{i}+{FP}_{i})}$$

$$Specificity=\frac{TN}{(FP+TN)}$$

$$Sensitivity=\frac{TP}{(TP+FN)}$$

$$F1=2*\frac{Precision*Sensitivity}{Precision+Sensitivity}$$

At this stage, to evaluate the performance of CNN against doctors by percentage agreement, three more orthopaedists and two radiologists who were completely unaware of this study participated in this study and independently classified the test group. A success ranking was determined after the results were obtained.

For statistical analysis, fleiss kappa, weighted kappa, and the intraclass correlation coefficient using the SPSS software pack (version 21, IBM, New York, USA) were used. *P* value < 0.05 was considered for statistical significance^31–34^.

### Hardware and software used in the experimental studies

Experimental studies carried out within the scope of this study were executed using an AMD Athlon X940 Black Edition processor, 16 GB of DDR3 RAM, and MSI GTX 1070 graphics card. In experimental studies, Python 3.6.5 was used as a programming language. In studies where Keras libraries are used to create DL architectures, Keras and Numpy libraries are used for image preprocessing. Evaluation metric values are calculated by authors using accuracy, precision, specificity, sensitivity, and F1 score equations.

### Ethics and consent to participate

The study protocol was approved by the institutional review board of the local ethics committee(No 15-01-2020/003). No patient consent was required because of the retrospective nature of the study. Patient data were anonymized in the paper.

## Results

According to the classification result, the confusion matrix shown in Table 2 is created. All accuracy, precision, sensitivity, and specificity results for every class and the whole system were calculated with the information given in the confusion matrix. In the confusion matrix in Table 2, the columns represent the classification results performed by the model, while the rows represent the ground truths of the data. Accuracy, Precision, Specificity, Sensitivity, F1 Score evaluation metrics represent the values obtained for each class, respectively, and the values in the last column represent the total result. The reason for using evaluation metrics other than Accuracy is to evaluate the performance of the model in each class separately and to prove that the model is not inclined to recognize a class.

**Table 2:** The Confusion Matrix and Evaluation Metrics of CNN Classification.

Predicted classes	A	B	C	Total
A	27	1	1	29
B	8	18	5	31
C	1	3	17	21
Total	36	22	23	81
Accuracy	0.7654	0.7654	0.7654	**0.7654**
Precision	0.75	0.8182	0.7391	0.7691
Specificity	0.8269	0.92	0.9	0.8823
Sensitivity	0.931	0.5806	0.8095	0.7737

Significant values are in bold.

The most successful model accuracy rate is 83.33% on validation, 76.54% on test images, according to the consensus list, and approximately 99.5% on training. Other precision, specificity, sensitivity, and F1 score results are shown in Table 2.

The results of six orthopaedists and CNN were analyzed using the Fleiss Kappa coefficient for the total and weighted Kappa between individual observers. Interobserver reliability was determined.

The ICC values of each orthopaedist were above 0.8, indicating good or excellent reliability. The ICC of CNN was 0.868, which is one of the highest in the literature. The kappa value for interobserver reliability between CNN and orthopaedists was 0.433–0.607 (Table 3).

**Table 3:** A summary of weighted k values and ICC Weighted kappa values less than 0.20 indicate poor agreement, 0.21 to 0.40 indicate fair agreement, 0.41 to 0.60 indicate moderate agreement, 0.61 to 0.80 indicate good agreement, and greater than 0.80 indicate very good agreement. ICC below 0.5 is considered poor reliability, 0.5 and 0.75 as moderate reliability, 0.75–0.9 as good reliability, and over 0.90 as excellent reliability^31,32^.

	DR2	DR3	DR4	CNN	DR5	DR6	Avg. ICC	95% CI	ICC rating
DR1	**0.825**	0.556	0.572	**0.607**	0.581	0.555	0.932	0.894–0.956	Excellent
DR2		0.547	0.642	0.605	0.626	0.598	0.929	0.890–0.954	Excellent
DR3			0.569	0.513	0.561	0.610	0.831	0.725–0.894	Good
DR4				0.478	0.732	0.783	0.859	0.656–0.930	Good
CNN					**0.433**	0.436	0.868	0.796–0.915	**Good**
DR5						0.774	0.853	0.744–0.912	Good
DR6							0.863	0.676–0.931	Good

Significant values are in bold.

We found that the overall Fleiss Kappa was 0.532, which indicates moderate agreement among all the reviewers, including CNN. Group A-B-C kappa scores separately were 0.677–0.354–0.565, respectively. The highest scores for the interobserver agreement were between DR1 and DR2, and between DR1 and consensus. CNN had the third highest agreement with the consensus among six orthopaedists (Table 4).

**Table 4:** Fleiss Kappa among all reviewers. 0.41–0.60 is considered moderate, 0.61–0.80 is considered substantial, and more than 0.80 is considered excellent^33,34^.

Rating category	Fleiss Kappa	*p*-value	Lower 95% CI	Upper 95% CI
A	0.677(s)	0.0005	0.636	0.718
B	0.354(p)	0.0005	0.313	0.395
C	0.565(m)	0.0005	0.524	0.607
Overall	0.532(m)	0.0005	0.503	0.561

To compare CNN's success to that of doctors, the rate at which images were correctly labeled by doctors based on the consensus list was compared to CNN's success rate. CNN's classification success rate was greater than 9 out of 11 doctors. CNN determined 62 of 81 (76.5%) images to be true. If we take them separately, CNN was achieved to determine 27/29 (93.1%) in group A, 18/31 (58.1%) in group B, and 17/21 (80.9%) in group C (Table 5).

**Table 5:** Quantitative evaluation of all assessors and CNN.

	A	B	C	Total
Dr 1	28	23	17	68 (84%)
Dr 2	25	25	18	68 (84%)
CNN	**27(93,1%)**	**18(58,1%)**	**17(80,9%)**	**62 (76,5%)**
Dr 3	21	17	20	58 (71,6%)
Dr 4	19	17	21	57 (70,3%)
Dr 5	22	16	18	56 (69,1%)
Dr 6	19	16	20	55 (67,9%)
Dr 7	25	8	19	52 (64,2%)
Dr 8	23	12	17	52 (64,2%)
Dr 9	17	12	21	50 (61,7%)
Dr 10	22	18	15	55 (67,9%)
Dr 11	26	18	17	61 (75,3%)
Consensus	**29**	**31**	**21**	**81 (100%)**

Significant values are in bold.

**Figure 5:**
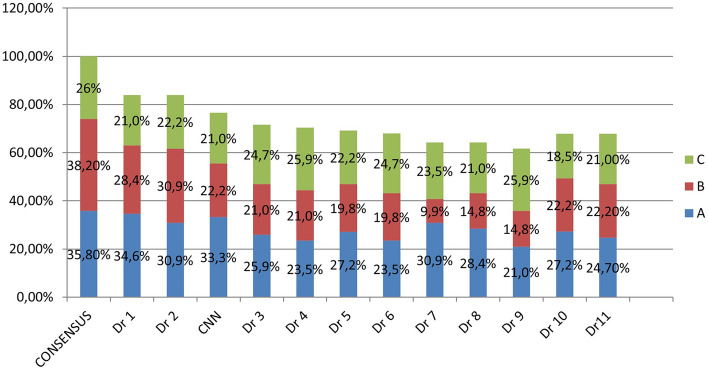
According to the consensus list, CNN achieved a %76.5 total classification performance. Only Dr1 (%84) and Dr2 (%84) had more achievements than CNN. CNN surpassed 9 of 11 doctors.

The distribution of LPC obtained by each doctor and CNN network is given in detail in Fig. 5.

## Discussion

In this study, LPC was performed using DL algorithms. Controversial results regarding the interobserver reliability of the LPC have been reported in the literature. Therefore, a DL algorithm was developed that can help orthopedists first. Then, reliability and performance tests were carried out. In this context, hip radiographs representing various stages of LCPD were created using the aforementioned method, and the CNN model was trained using these radiographs. The model, whose training was completed, was tested using 81 hip radiographs belonging to real patients. Eleven doctors' test results were compared with the CNN model's results.

Several clinical studies of CNNs have been proposed in the medical sciences since 2012. The initial AI applications in medicine have usually focused on image recognition tasks, such as diabetic retinopathy, mammographic lesions, and skin cancer recognition. With varying degrees of success, CNN was used in orthopedics for recognition and segmentation tasks such as fractures of various bones, meniscal tears, knee cartilage lesions, anterior cruciate ligament tears, vertebral body localization and segmentation, bone age assessment, and degenerative spine interpretation^35^. While various DL models demonstrate expert-level performance even now, they also have the potential to exceed expert physicians in the future. Olczak et al.^36^ compared five openly available DL networks to identify fracture, laterality, body part, and exam view and published at least 90% accuracy for laterality, body part, and exam view and 83% accuracy for fracture identification. Chung et al.^37^ trained a deep CNN to detect and classify the proximal humerus fractures using 1891 images. They concluded that CNN showed superior performance over general physicians and orthopedists and similar performance to orthopedists specializing in the shoulder. In complex 3- and 4-part fractures, CNN showed superior performance. Helm et al.^38^ reviewed the literature and reported that AI helps orthopedic surgeons better serve their patients. Urakawa et al.^39^ reported in their study that AI was better than five orthopedists for detecting hip fractures. In their systematic review, Langerhuizen et al.^40^ reported that AI reflected near-perfect prediction for fracture detection in five studies. AI outperformed human examiners for the detection and classification of hip and proximal femur fractures. One study showed equivalent performance for wrist, hand, and ankle fractures.

The accuracy and precision of our CNN model were 76.54% and 76.91%, respectively, compared to the 62% accuracy and 52% precision result in the support vector machine classifier, according to the Waldenstrom classification of LCPD^20^.

LPC has some advantages, including reproducibility, ease of application (requires only an AP radiograph), and high correlation with the outcome. According to many authors, LPC has the highest interclass reliability among the three classification systems^6–11^.

Recent studies demonstrated highly variable reliability; the ICC for LPC was reported as 0.388–0.596^12^, and as 0.70–0.85^41^, so the ICC of CNN in this study was one of the highest previously reported. CNN's weighted kappa was compatible with previously reported values of 0.722^7^, 0.71–0.79^41^, 0.65–0.70^42^, 0.56–0.70^43^, and 0.510^9^.

Many authors studied the prognostic value of this classification, and a high association was found with the final outcome^2–5^. Patients with Group A and Group B below eight years of age are treated symptomatically; patients with Group C and the Group B/C border above eight years of age need surgical containment. However, there is a debate at this point. Some authors thought that there were changes in the groups between the first and last classifications. Lappin et al.^11^ stated that 75% of group A and 30% of group B cases required upgrading within seven months of initial symptoms. Meurer et al.^14^ reported the need for upgrading in 30% of cases. Park et al.^10^ stated that 45% of cases needed upgrades between their initial and final grades. Thus, the initial and final grades of the hip can be different, and this difference may postpone the containment treatment to preserve the shape of the femoral head, especially in patients under eight years of age. Even Akgun et al.^40^ suggested the use of measurements instead of estimates, especially in borderline cases when LPC was to be used as the prognostic indicator. We predict that CNN will be a more accurate predictive indicator in terms of classification and predicting prognosis, especially in the future, as underlined by Price^15^.

We have some limitations in our study. First, test and training data should never be mixed to accurately assess the CNN's performance. When similar studies in the literature were examined, it was seen that many real patient images were used in the studies. Chung et al.^37^ used 515 normal and 1376 fractured proximal humerus images for this purpose. Urakawa et al.^39^ used a total of 3346 hip images to train CNN in their study. Even considering that the incidence of LCPD ranges from 0.4–29/100,000 children < 15 years of age^44^, the probability of finding data close to the numbers in the mentioned studies is almost negligible in a two-center study. Thus, we did not have enough labeled radiographs to use them separately to execute both the test and training. We designate such a method to overcome this problem. The images, which were obtained by modifying normal hip radiographs, were used only to train CNN, while real patients' images were used only to test. Secondly, by our method, we only changed the height of the femoral head, especially in the lateral pillar. Femoral head fragmentation seen in the natural course of LCPD was not present in our images obtained from normal hip radiographs. Although we believe that this disadvantage may have negatively affected our results, our CNN still showed better performance than 9 out of 11 doctors, which is a very promising result. We believe if we had the same number of real patient radiographs as we produced to train, the performance of CNN could surpass us all. In addition, our study demonstrates that it is possible to train CNN and obtain good results using the data produced by the aforementioned method, particularly in studies where patient-specific data is insufficient.

## Conclusion

It is widely known that large quantities of data are used during the training of CNN to ensure successful outcomes. This research presents a novel method for training deep neural networks in the presence of limited data, such as that associated with rare medical conditions like LCPD, by employing modified data during training. AI systems can be employed for many rare diseases with this technique, in which only healthy data is utilized by modifying it. Moreover, this is the first study to perform LPC on x-ray images of LCPD patients. The results indicate that the classification accuracy of the developed model is superior to that of many of the doctors who participated in the study. Since the modified data is constructed with a predetermined pattern structure, we predict that the real pattern structure will yield superior results, as it will reflect not just the changes in head height, but also other structural alterations that occur in the femoral head during the disease.

## Data Availability

The data used during the current study are available from the corresponding author on reasonable request.

## Abbreviations

LCPD: Legg–Calve–Perthes disease
LPC: Lateral pillar classification
AI: Artificial İntelligence
CNN: Convolutional neural network
DL: Deep learning
ML: Machine learning
ANN: Artificial neural network
MRI: Magnetic Resonance Imaging
ReLU: Rectified linear unit
TP: True positive
TN: True negative
FP: False positive
FN: False negative
ICC: Intraclass correlation coefficient
